# Squamous Cell Carcinoma and Its Rare Variant, Carcinoma Cuniculatum: Insights and Case Studies

**DOI:** 10.3390/cancers17071217

**Published:** 2025-04-03

**Authors:** Klaudia Knecht-Gurwin, Aleksandra A. Stefaniak, Iwona Chlebicka, Lukasz Matusiak, Zdzisław Woźniak, Jacek C. Szepietowski

**Affiliations:** 1 University Centre of General Dermatology and Oncodermatology, Wroclaw Medical University, 50-556 Wroclaw, Poland; aleksandraannastefaniak@gmail.com; 2 Division of Dermatology, Venereology and Clinical Immunology, Faculty of Medicine, Wroclaw University of Science and Technology, 51-377 Wroclaw, Poland; iwonak4wsk@interia.pl (I.C.); luke71@interia.pl (L.M.); 3 Department of Dermato-Venereology, 4th Military Hospital, 50-981 Wroclaw, Poland; 4 Department of General and Experimental Pathology, Wroclaw Medical University, 50-556 Wroclaw, Poland; zwozniak9@wp.pl

**Keywords:** squamous cell carcinoma, carcinoma cuniculatum, epithelioma cuniculatum, verrucous carcinoma, keratopapilloma

## Abstract

Background: Squamous cell carcinoma (SCC) presents a significant challenge in dermatological pathology, necessitating comprehensive insights for effective management. This review highlights SCC’s broad aspects and focuses particularly on carcinoma cuniculatum (CC), a rare variant that warrants special attention due to its unique diagnostic and therapeutic challenges. Methods: We conducted a narrative review of current literature on SCC and CC, supplemented by illustrative clinical case presentations. The analysis emphasized epidemiology, pathogenesis, clinical presentation, diagnostic pitfalls, and treatment modalities. Results: The key findings from our analysis indicate that CC, although locally aggressive, exhibits a lower metastatic potential and requires distinct management strategies, primarily surgical, due to its poor responsiveness to radiotherapy. Conclusions: The review also includes illustrative clinical cases to highlight the diverse presentations and underscore the necessity for multidisciplinary approaches and heightened clinical vigilance in diagnosing and managing CC. By consolidating current knowledge and clinical experiences, this review aims to enhance awareness, facilitate early recognition, and optimize therapeutic strategies for SCC, particularly CC, thereby improving patient outcomes and prognosis.

## Simple Summary

Squamous cell carcinoma (SCC) is a common skin cancer, with carcinoma cuniculatum (CC) representing a rare subtype. CC predominantly affects the soles of the feet and is associated with chronic trauma and inflammation rather than UV exposure. It presents as slow-growing, wart-like lesions with keratin-filled tracts, often leading to misdiagnosis as benign conditions such as verruca vulgaris. While CC is usually well-differentiated and rarely metastasizes, it can cause significant local destruction if untreated. Accurate diagnosis is essential to avoid delays in treatment. Surgical removal remains the primary treatment modality, with wide local excision (WLE) and Mohs micrographic surgery (MMS) being the most effective options. This review aims to improve the recognition and management of CC by summarizing its unique clinical features, diagnostic challenges, and treatment strategies, offering practical insights for clinicians handling this uncommon but impactful disease.

## 1. Introduction

Squamous cell carcinoma (SCC) stands as one of the most prevalent types of malignancies worldwide, encompassing a spectrum of histopathological variants [1]. Among these variants, carcinoma cuniculatum (CC) emerges as a distinct entity, characterized by its unique clinical and histological features. While SCC has been extensively studied, CC remains relatively rare and poorly understood, posing challenges in diagnosis and management. First described in 1954, CC is a locally aggressive, slow-growing SCC variant with distinct exophytic clinical features and well-differentiated histology. Notably, CC primarily affects the plantar surface of the foot, presenting diagnostic and therapeutic challenges to clinicians. Despite its distinct clinical and histopathological attributes, the precise etiology and pathogenesis of CC remain incompletely understood. Given its rarity and specific clinical characteristics, understanding the nuances of CC is essential for accurate diagnosis, optimal management, and improved patient outcomes [2,3].

This study embarks on a comprehensive exploration of SCC, shedding light on its multifaceted nature while delving into the intricacies of CC. By amalgamating clinical observations, histopathological analyses, and therapeutic considerations, we aim to elucidate the distinctive aspects of CC within the broader context of SCC.

## 2. Epidemiology

SCC is the second most prevalent skin malignancy, constituting approximately 20% of all cutaneous cancers [4]. Its incidence is closely linked to age, with a mean onset at around 70 years old, and it is observed to occur twice as frequently in males as in females. Furthermore, environmental and geographic factors, notably latitude and ultraviolet (UV) radiation exposure, play a pivotal role in its prevalence. Regions with intense sunlight exposure, such as Australia, report the highest incidence rates, whereas in Europe and the United States, the incidence is estimated at 77 and 262 cases per 100,000 person-years, respectively. Individuals with fair skin (Fitzpatrick phototypes I and II), who possess reduced melanin-mediated UV protection, are disproportionately affected [5].

CC, a rare and distinctive variant of SCC, is predominantly found on the plantar surfaces of the feet and is strongly associated with chronic trauma and persistent inflammatory states. Unlike conventional SCC, CC exhibits a markedly lower incidence and presents with a unique demographic profile, typically affecting individuals in their third to eighth decades of life. Moreover, its development is often linked to pre-existing lesions, such as diabetic foot ulcers, verrucous hyperplasia, or chronic wounds. Be that as it may, CC remains under-recognized, which contributes to delayed diagnosis and suboptimal management [4,6].

## 3. Clinical Presentation

Clinically, SCC manifests with diverse morphologies, ranging from scaly, erythematous plaques to ulcerated or hyperkeratotic nodules. These lesions are frequently observed on sun-exposed areas, such as the face, ears, and hands, and tend to exhibit rapid growth coupled with localized tissue destruction. Notably, high-risk cSCC lesions often demonstrate features such as a diameter exceeding 2 cm, poorly defined margins, or perineural invasion, all of which are associated with a significantly elevated risk of recurrence and metastasis [7].

CC, by contrast, presents as a slow-growing, exophytic lesion characterized by a verrucous, cauliflower-like appearance. A hallmark of CC is the presence of keratin-filled sinus tracts, often accompanied by malodorous discharge. Owing to its indolent nature, CC is frequently misdiagnosed as benign conditions, such as verruca vulgaris or hyperkeratosis, resulting in significant diagnostic delays. Unlike conventional SCC, CC is generally well-differentiated and exhibits a lower propensity for metastasis; however, it can lead to substantial local tissue destruction if left untreated [8].

Differentiating CC from other conditions, such as verruca vulgaris, keratoacanthoma, or amelanotic melanoma, remains a formidable diagnostic challenge (Table 1). Moreover, the indolent course of CC, coupled with its occurrence in UV-protected areas, necessitates heightened clinical vigilance. Consequently, timely recognition and accurate diagnosis are paramount to mitigating delays and ensuring appropriate therapeutic interventions.

## 4. Pathogenesis

Prolonged sun exposure, especially during childhood and adolescence, is the primary risk factor for SCC [24,25]. Furthermore, in recent times, immunosuppression, which includes that related to organ transplantation, has emerged as an escalating contributor to tumorigenesis, and the development of SCC in regions of persistent inflammation should not be disregarded [26]. Over the past several years, it has been determined that types of human papillomaviruses (HPV), contributing to genital disease manifestations, are comprehended as carcinoma elicit factors [27]. Additionally, it has been suggested that cutaneous beta-HPV (b-HPV) strain types may have a role in the pathogenesis of SCC [28].

It is consistent with the fact that in epidermodysplasia verruciformis patients, there is a heightened risk of SCC development, as well as increased detection of b-HPV types in SCC equally in immunosuppressed and immunocompetent individuals. Typically, cutaneous variants of the virus, including the most common types such as HPV 2, 57, and less frequent HPV 1, 4, 7, are not correlated with the development of SCC, although there are some reports that confirm such an association. It appears that b-HPV infection exerts a significant role in initiating carcinogenesis, albeit this may not be necessary for tumor maintenance [29]. This supposition is substantiated by the observation of an elevated viral load in premalignant lesions like actinic keratoses, relative to SCC [30]. Nevertheless, the substantial prevalence of b-HPV types in healthy skin implies the influence of additional cofactors in the pathogenesis of SCC. It is proposed that chronic inflammation serves as a pivotal contributor to b-HPV-associated carcinogenesis. Existing literature on this subject strongly indicates that the role of b-HPV types should not be underestimated in the initiation of SCC [31]. Nevertheless, the role of HPV infection in SCC development remains alleged but should be taken into consideration as a risk factor in the combination of solar UV-induced radiation damage [27]. As far as CC is concerned, an association with human papillomavirus (HPV), notably subtypes 11 and 16, has been documented [32].

A rare variant of SCC known as verrucous carcinoma (VC), characterized by its infrequent occurrence and well-differentiated features, constitutes the particular focus of our investigation. This distinct subtype was first documented by Lauren V. Ackerman in the year 1948 [33]. The lesions often present as slow-growing plaques, ulcerations, or cauliflower-like tumors and occur in various regions, including the following [8]:

- Oral cavity (oral florid papillomatosis);
- Urogenital regions (giant condyloma acuminatum Buschke-Löwenstein);
- Surgical sites (amputation stumps);
- Sole of the foot (epithelioma cuniculatum, EC; also referred to as CC).

Epithelioma cuniculatum (EC) predominantly affects the plantar surface of the foot, with lesions localized as follows [34]:

- Ball of the foot: 53% of cases;
- Toes: 21%;
- Heel: 16%.

Clinically, CC manifests with infection-like features, including sinuous fistulas that discharge yellow, malodorous, keratinous material [35]. These lesions are often initially misdiagnosed as recalcitrant warts due to their persistent and wart-like appearance, leading to delays in treatment [36]. Although CC rarely metastasizes, it can invade adjacent soft tissue and bone, which may require extensive surgical resections or even amputation in advanced cases [37].

Histologically, CC exhibits the following characteristics [38]:

- Hyperkeratosis, papillomatosis, and acanthosis;
- Broad, blunt-ended rete ridges with a characteristic “bulldozing” architectural pattern;
- Minimal cellular atypia, though pleomorphic changes and eosinophilic cytoplasm may be observed, differentiating EC from common warts and conventional SCC.

Immunostaining with Ki-67 antigen and p53 can aid in distinguishing CC from other lesions, such as plantar verrucae and conventional SCC, especially when diagnosis proves challenging [38].

Subsequent to diagnosis, computed tomography or magnetic resonance imaging may be indispensable in delineating tumor extension and assessing for involvement of deep tissues and bone structures [39].

### SCC and CC Risk Factors

SCC is a multifactorial disease influenced by a complex interplay of various risk factors. Undoubtedly, cumulative ultraviolet radiation (UVR) exposure is the most significant environmental risk factor for SCC. Both UVB and UVA radiation induce DNA damage, leading to C-T and CC-TT dipyrimidine transitions, which represent the majority of mutations found in SCC [40]. UVA radiation’s impact is primarily indirect, as it generates reactive oxygen species (ROS) through photooxidative stress [41].

In addition to mutagenic effects, UVR facilitates SCC development by exerting immunosuppressive actions, including the following [42]:

- Depletion of Langerhans cells in the epidermis;
- Impaired antigen presentation in skin-draining lymph nodes;
- Hindered tumor surveillance due to the expansion of regulatory T cells;
- A shift towards T-helper type 2 responses.

In CC, however, UVR-driven mechanisms play a lesser role, as CC typically develops in UV-protected areas, such as the soles of the feet. Instead, chronic trauma and inflammation serve as the primary contributors to CC pathogenesis. Repeated pressure and irritation, particularly on weight-bearing areas, align with CC’s development. This mirrors SCC occurrence in chronically inflamed regions such as wounds or sinus tracts [43].

Additional Risk Factors:

1. Pharmacological treatments [44]:
  - Prolonged use of photosensitizing drugs, such as antibiotics, fluoroquinolones, and triazole antifungals, increases SCC risk;
  - Long-term UVA therapy for chronic skin conditions further exacerbates the risk, particularly in immunosuppressed individuals and those with sun-sensitive skin types.
2. Immunosuppression [45]:
  - Immunosuppressed patients, such as organ transplant recipients or individuals on long-term immunosuppressive therapy, show a marked increase in CC risk. This elevated risk likely results from impaired tumor surveillance and a higher incidence of chronic infections, including HPV.
3. Chronic inflammation and carcinogens [46]:
  - Occupational and environmental exposures to hydrocarbons, arsenic, or polycyclic aromatic hydrocarbons stimulate carcinogenic pathways, even without UV damage, underscoring the environmental contribution to CC pathogenesis.
4. Genetic factors:
  - Variations in genes involved in melanization (e.g., the melanocortin-1 receptor) are associated with increased UV sensitivity and SCC risk, particularly in individuals with fair skin [42,47].
  - Genetic skin disorders significantly elevate SCC risk, including the following [40,48]:
    ▪ Recessive dystrophic epidermolysis bullosa (RDEB) caused by COL7A1 mutations, leading to aggressive SCC;
    ▪ Other syndromes, such as Lynch syndrome, Muir–Torre syndrome, xeroderma pigmentosum, and Fanconi anemia, impair DNA repair and genomic stability, predisposing individuals to SCC.

For CC, the role of genetic factors remains less defined, as UVR is not a primary driver. However, unique genetic susceptibilities may predispose individuals to CC, particularly in areas subject to trauma or chronic inflammation [48].

**Figure 1:**
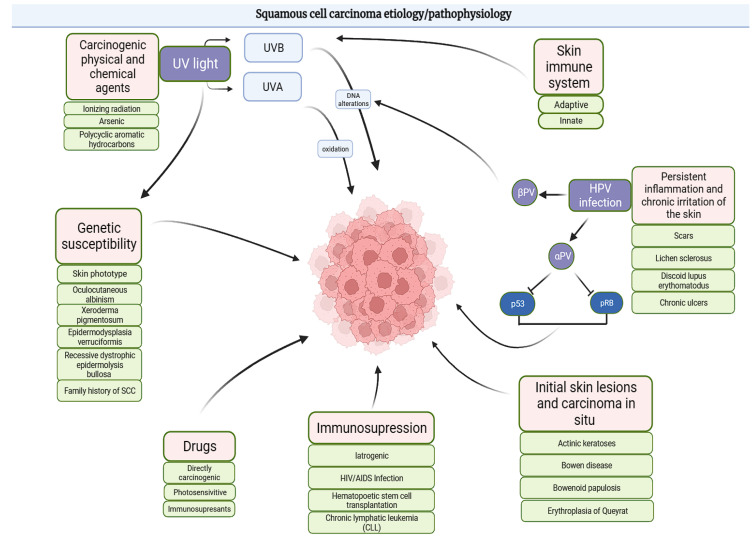
Risk factors of SCC development (Figure created with BioRender.com).

In summary, CC exhibits a multifaceted etiology within the broader context of SCC. While both share some pathophysiological aspects, CC’s pathogenesis is less reliant on UVR and more influenced by chronic trauma, HPV infection, environmental carcinogen exposure, and immunosuppression, underscoring the variant’s unique clinical and histological features. All the aforementioned risk factors are subsumed in Figure 1.

## 5. The Role of HPV Infection

The presence of certain types of HPV constitutes a recognized predisposing factor for the onset of anogenital SCC. However, the development of SCC, including CC, in the context of viral alterations, excluding the anogenital region, is not straightforward; nonetheless, an increasing body of literature points towards the involvement of beta HPV subtypes [49]. Cutaneous beta-HPVs lack DNA integration ability but may interfere with cellular DNA repair or apoptosis, enhancing susceptibility to UV-induced damage. Beta-HPV E6 proteins, by inhibiting UV-induced apoptosis and activating telomerase, contribute to oncogenicity by impairing p53 tumor-suppressor function [49,50]. UV light transiently suppresses skin immunity, increasing susceptibility to HPV infection. Immunocompromised patients exhibit higher HPV prevalence in SCC compared to immunocompetent individuals [27].

**Figure 2:**
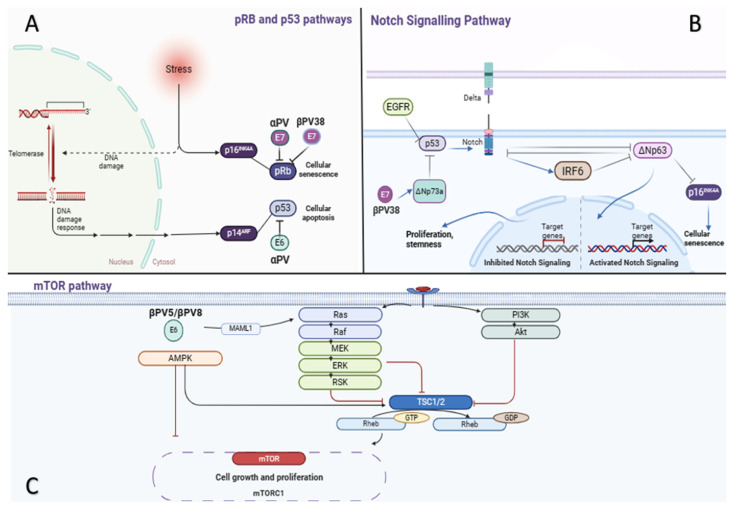
Pathways involved in SCC pathogenesis (Figure created with BioRender.com): (**A**) High-risk alpha human papillomaviruses (alphaPV) inhibit p53 and retinoblastoma (pRb) through E6 and E7 oncoproteins, respectively, disrupting cell cycle regulation, senescence, and apoptosis. Beta human papillomaviruses (betaPV), like BetaPV38, promote procarcinogenic effects by enhancing UV-induced DNA damage accumulation in the skin. (**B**) Notch pathway inactivation, induced by EGF-R activation, p53 inactivation, or NOTCH gene mutations, favors cell proliferation, survival, and stemness, while hindering senescence and apoptosis. Upregulated ΔNp63 represses HES1, p21, and p16INK4A. HPV38 E7 induces DNp73a, a p53 antagonist, inhibiting the expression of p53-regulated genes. (**C**) The RAS-RAF-MEK-ERK and PI3K/AKT/mTOR pathways, activated by RTK and RAS, promote cell proliferation and survival. Mutations in RTK or RAS and RASA1 inactivation lead to constitutive activation. PI3K/AKT activation also contributes to pathway activation. Increased PI3K signaling reduces TSC complex activity, releasing its inhibition on mTORC1, facilitated by Rheb. This leads to heightened mTOR1 catalytic activity. BetaPV5 and -8 E6 proteins recruit MAML1, suppressing Notch signaling, leading to a disruption in the balance between Notch and RAS, favoring RAS. Upregulated signaling promotes cell proliferation over differentiation.

In both SCC and CC, pathogenesis involves intricate molecular pathways that drive the development of this malignancy. These pathways encompass crucial cellular processes, including cell cycle regulation, apoptosis, senescence, differentiation, and mitogenic/survival pathways (Figure 2). The tumor suppressor genes p16INK4A and p14ARF play significant roles in controlling the pathways. The former controls the retinoblastoma (pRb) pathway, the latter, the p53 pathway. Their loss of function disrupts the cell cycle, impeding senescence or apoptosis [51]. The carcinogenic mechanisms of high-risk alpha human papillomaviruses (alphaPV) in anogenital cancer have been extensively investigated and entail well-defined pathways, characterized by the inhibitory action of viral E6 and E7 oncoproteins on the tumor suppressors p53 and retinoblastoma, respectively [52]. Beta human papillomaviruses (betaPV) exhibit distinct patterns, likely promoting procarcinogenic effects primarily through the facilitation of UV-induced DNA damage accumulation in the skin. In normal cellular environments, ultraviolet (UV) radiation stimulates the upregulation of cellular defense mechanisms, culminating in the activation of p53, cell-cycle arrest, apoptosis, or DNA repair processes. For instance, Beta human papillomavirus 38 (BetaPV38) E7 protein has the capability to bind and degrade pRb to a level comparable to that of the AlphaPV16 E7 protein [28]. Significantly, the involvement of beta-HPVs in CC could be linked to their ability to utilize non-UV related pathways during carcinogenesis. In cases of CC, it is suggested that beta-HPVs, notably HPV 5 and 8, might influence keratinocyte regulatory mechanisms without the influence of UV light exposure. Therefore, the alterations caused by beta-HPV in the tumor suppressor pathways, notably via the actions of E6 and E7 proteins on p53 and pRb, are thought to promote the pronounced local expansion and limited metastatic tendency of CC, which sets it apart from the generally more aggressive variants of SCC [31].

Furthermote, aberrant activation of the epidermal growth factor receptor (EGF-R), inactivation of p53, or mutations in the NOTCH gene lead to the inactivation of the NOTCH pathway. Consequently, the suppression of ΔNp63, facilitated either directly or through IRF6, is compromised, promoting cell proliferation, survival, and stemness. The inactivation of NOTCH also hinders senescence and apoptosis [53]. Additionally, upregulation of ΔNp63 leads to the repression of p16INK4A. Moreover, the HPV38 E7 protein induces the accumulation of DNp73a, a p53 antagonist, which subsequently inhibits the expression of several genes regulated by p53 [54]. Emerging evidence indicates that the E6 proteins of BetaPV5 and BetaPV 8 possess the ability to recruit MAML1 (Mastermind-like protein 1), leading to the suppression of the cutaneous tumor-suppressive Notch signaling pathway [55]. Consequently, a disruption in the equilibrium between Notch and the oncogene RAS occurs, favoring RAS. The abrogation of Notch signaling also triggers the up-regulation signaling in keratinocytes, representing a well-known pathway in skin carcinogenesis that is likely to promote cell proliferation at the expense of differentiation [56].

The RAS-RAF-MEK-ERK-RSK and PI3K/AKT pathways exhibit overlapping upstream protein constituents, such as tyrosine kinase receptors (RTK) and RAS. Activating mutations in RTK or RAS, as well as the inactivation of the negative regulator RASA1, result in constitutive activation of both pathways, promoting cell proliferation and survival. Abnormal activation of these pathways may also occur due to PI3K/AKT activation, or inactivation of Phosphatase and tensin homolog (PTEN) [51]. Increased PI3K signaling reduces TSC complex activity, comprising TSC1/2, which normally inhibits mTORC1 [57]. This modulation involves Rheb, a GTPase, binding to mTORC1 to enhance its catalytic function [58]. Several drugs targeting RTKs and downstream pathways can be employed to inhibit SCC progression. However, it is noteworthy that RAS mutations, commonly found in photodamaged skin, may activate both pathways as part of a compensatory mechanism, potentially leading to resistance against therapeutic targeting strategies [51,59].

## 6. Clinical Cases

### 6.1. Clinical Case 1

**Figure 3:**
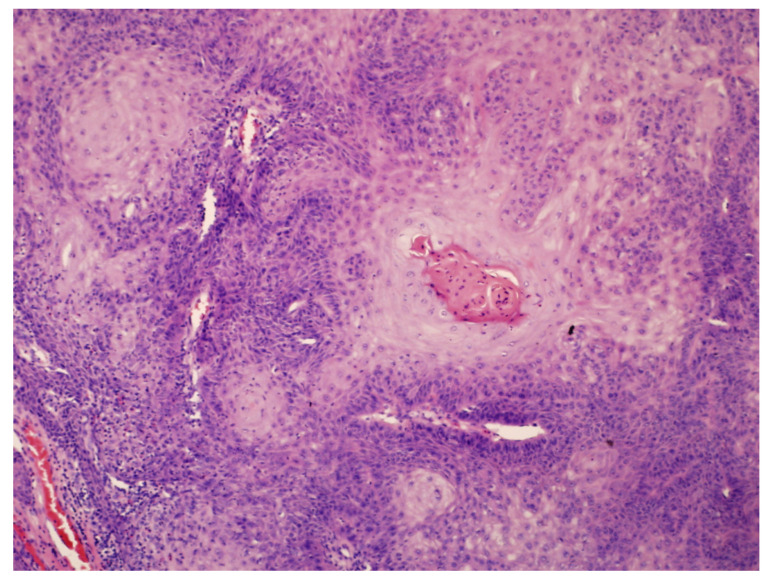
A central keratin pearl within well-differentiated squamous cell carcinoma nests (H&E; original magnification ×100).

**Figure 4:**
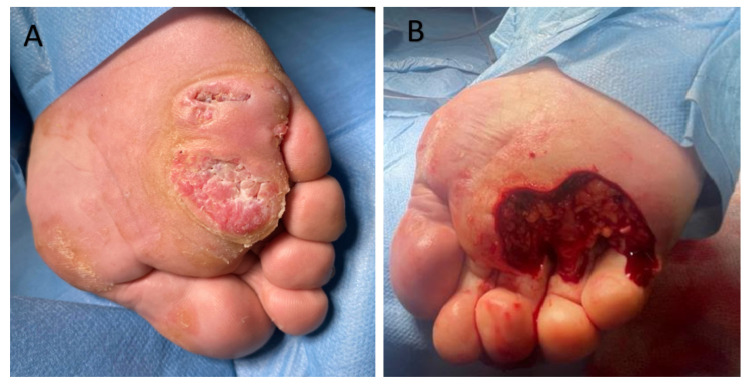
A male patient with verrucous carcinoma of the anterior foot. (**A**) The verrucous extensive lesion is distinctly demarcated from the surrounding area by a yellowish border. (**B**) Status post-tumor resection with a 5 mm margin.

A 72-year-old male patient was admitted to the Dermatological Clinic for surgical excision of a tumor on the sole of his right foot. The patient claimed to have observed the initial alterations such as wart-like lesions approximately 2 years prior. On physical examination, a hyperkeratotic and tumorous exophytic lesion measuring 50 mm in diameter was noted in the anterior plantar region. The patient exhibited palpable pedal pulses, and motor function remained unimpaired. There were no evident bony prominences or significant deformities observed. Previous treatment included fluorouracil and two surgical excisions, each procedure resulting in recurrence and enlargement of the lesion. A punch biopsy was performed. Upon histopathological examination, the characteristic blunt papillary projections of a well-differentiated squamous cell carcinoma with minimal nuclear pleomorphism and conspicuous keratinization, were observed (Figure 3). The diagnosis of CC was established. During the patient’s hospitalization, the tumor was surgically excised with a 5 mm margin, along with the removal of infiltrating nodules in multiple elusive-to-detect sinus tracts, penetrating the intermetatarsal space of the anterior foot. After achieving granulation tissue, the wound was covered with a skin graft, which healed without complications (Figure 4).

### 6.2. Clinical Case 2

**Figure 5:**
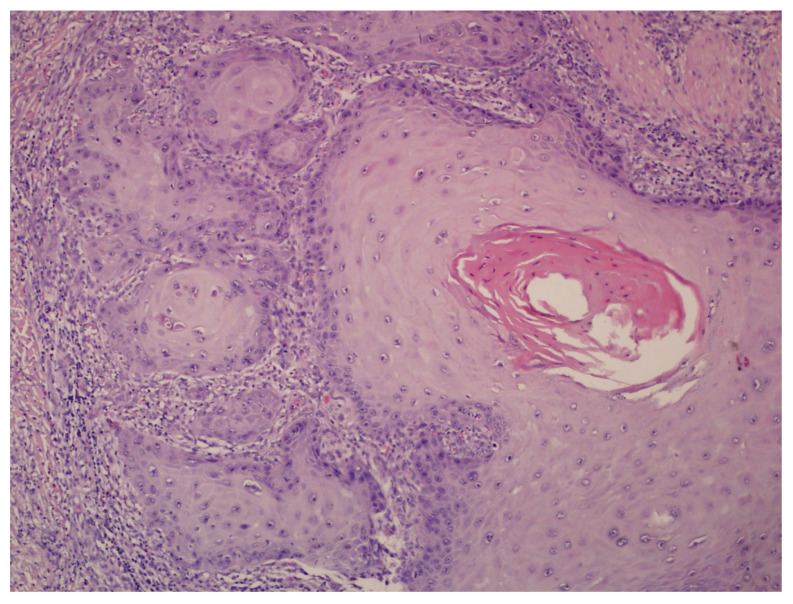
Well-differentiated squamous cell carcinoma with a central, prominent keratin pearl formation, surrounded by invasive tongues forms component and inflamed associated stroma (H&E; original magnification ×100).

**Figure 6:**
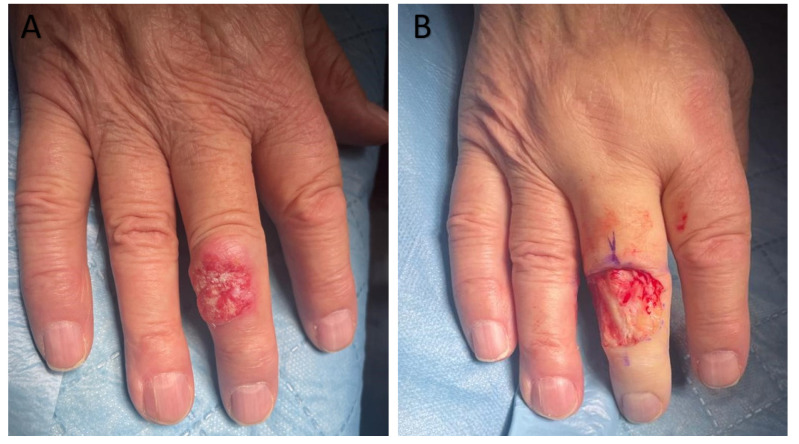
The tumor on the dorsal surface of the third finger of the left hand, at the level of the proximal interphalangeal joint. (**A**) Raised, irregularly shaped, and thickened mass with a crusted surface situated on reddened background. (**B**) The wound after the excision of the lesion with a margin of healthy tissues.

A 30-year-old woman was admitted to the local clinic for scheduled diagnostic and therapeutic purposes regarding a skin lesion. Upon admission, a thick plaque covered with crusts was observed on the dorsal surface of the third finger of the right hand. The skin lesion is painful upon palpation and tends to bleed even with minor trauma, which prevents complete healing of the surface wound. The initial appearance of the eruption occurred approximately four years before the admission without any identifiable triggering factor, according to the dermatologist’s report from the outpatient clinic. The morphology of the lesion resembled a viral wart. Approximately six months prior, an ineffective cryotherapy procedure was performed, and local preparations were used in the treatment, the names and compositions of which are unknown, but without clinical improvement. Over the two months, the eruption had started to enlarge and change in consistency, becoming harder. Furthermore, it had become poorly movable relative to the underlying surface. The patient displayed detectable pulses, with intact motor function. No apparent bony protrusions or notable deformities were identified during examination. The lesion was surgically excised using the Mohs micrographic surgery technique, reaching the extensor tendon sheath. The resulting defect was covered with an intermediate-thickness skin graft. The differential diagnosis included lupus vulgaris and verruca vulgaris. However, histological examination revealed broad, and bulbous papillary projections composed of well-differentiated SCC with minimal cytological atypia (Figure 5). Moreover, prominent inflammatory infiltrate in the stroma surrounding the tumor was noted. The diagnosis of CC was confirmed. No recurrence was observed in the 6-month follow-up (Figure 6).

### 6.3. Molecular Pathway Speculation in Carcinoma Cuniculatum Case Studies

In the two case studies of CC presented, the notable local invasiveness observed may be underpinned by specific molecular pathways that govern cellular behavior. The disruptions in the Notch signaling pathway could contribute significantly to the unchecked growth and local spread of CC, given its role in cell differentiation and proliferation [54]. Additionally, the involvement of the RAS-RAF-MEK-ERK signaling pathway, a critical cascade in the regulation of cell growth and survival, may also play a pivotal role [51]. This pathway is often activated in various cancers and could be contributing to the extensive local invasion seen in CC by driving further cellular proliferation and migration. These molecular interactions, particularly in areas subjected to chronic mechanical stress such as the soles of the feet, might synergistically exacerbate the tumor’s invasive properties. Speculating on these pathways provides a crucial understanding necessary for the development of targeted therapies aimed at controlling the local invasiveness of CC, ultimately improving patient outcomes by preserving local tissue integrity and function.

## 7. Treatment

CC is a distinctive variant of low-grade SCC known for its locally aggressive growth pattern and proclivity for slow metastasis. The primary therapeutic modality for this neoplasm entails singular radical surgical excision, which constituted the predominant treatment approach in the majority of cases [60]. Of the various surgical techniques employed, wide local excision (WLE) was the most frequently reported. Incidence of recurrence after WLE is observed in about half of cases, with repeat excision boasting wider margins or amputation being the preferred secondary treatment for recurrent occurrences [6].

Amputation, while less commonly utilized, emerged as the second most prevalent treatment modality, exhibiting a high incidence of recurrence [6]. It may be accompanied by complications such as infection and the necessity for prosthetic assistance for ambulation [61].

In contrast, Mohs micrographic surgery (MMS), being the least frequently reported surgical intervention, featured horizontal en face sectioning of tissues. Notably, recurrence rates following MMS were observed in the majority of cases. In those cases, repeated MMS proved valuable, with some necessitating additional removal of tumors entwined around anatomical structures like the flexor tendon [6,62].

Systemic therapy utilizing agents like acitretin and interferon alfa was seldom employed. Additional therapeutic modalities, including imiquimod, curettage, debridement, electrodesiccation, and photodynamic therapy, exhibited infrequent application [63,64].

Moreover, the dysregulation of the RAS-RAF-MEK-ERK signaling pathway plays a notable role in the pathophysiology of CC and SCC, impacting cell proliferation and survival. Mutations in the RAS or RAF genes often activate this pathway persistently in various tumors, including CC, which leads to enhanced cell growth and a tendency for invasion. Given the complexity and the critical role of this pathway in CC, targeted therapies that inhibit components of the RAS-RAF-MEK-ERK cascade represent a promising area for developing more effective treatments. Such targeted therapies might include MEK inhibitors, which have shown potential in other cancers with similar pathway dysregulation [65].

While prophylactic lymph node dissection was generally deemed unnecessary given the rarity of lymph node involvement, instances of focal malignancy within the verrucous carcinoma, particularly in the oral and genital regions, were reported. Consequently, in cases with a heightened risk of metastasis, prophylactic lymphadenectomy was deemed justified [66,67].

In summary, the management of verrucous carcinoma hinges on surgical intervention as the principal therapeutic approach. Wide local excision, being the most frequently employed procedure, is complemented by alternatives such as Mohs micrographic surgery and amputation in select cases. Systemic therapy and other treatment modalities are utilized sparingly. The judicious consideration of prophylactic lymph node dissection is typically reserved for high-risk scenarios, emphasizing the significance of appropriate surgical intervention and vigilant post-treatment monitoring to achieve favorable outcomes for patients afflicted with verrucous carcinoma.

## 8. Conclusions

SCC and its rare variant, CC, exhibit distinct etiologies and clinical challenges. While SCC typically develops in sun-exposed areas due to ultraviolet (UV) radiation, CC commonly arises in non-sun-exposed regions, such as the soles, and is strongly associated with chronic trauma and HPV infections. Both SCC and CC involve complex molecular pathways, including alterations in p53 and NOTCH signaling, yet CC poses unique diagnostic difficulties due to its resemblance to benign lesions. Effective management often involves surgical excision, and clinical cases highlight the diverse presentations and specific therapeutic approaches necessary for optimal treatment of CC.

## Figures and Tables

**Table 1:** Subsumed medical conditions encompassing the differential diagnosis of carcinoma verrucosum, juxtaposing the clinical presentation, histological examination, and dermatoscopic features.

Differential Diagnosis	Clinical Features	Histological Findings	Dermoscopic Findings	Sources
Refractory Verruca Plantaris	Plaque with hyperkeratotic surface painful under pressure Numerous black dots on the surface Lesions may coalesce into a “paving stone” pattern	Hyperkeratosis, acanthosis, koilocytosis, papillomatosis	Mosaic pattern with papillae surrounded by haloes Interrupted skin lines and brownish background	[9,10]
Keratoacanthoma	Rapidly growing dome-shaped nodules with central keratin plug	Well-differentiated squamous cells Keratin-filled plug	Keratin-filled invagination White keratin surface crown vessels	[11,12]
Eccrine Poroma or Porocarcinoma	Solitary, firm, slow-growing, dome-shaped papule or nodule skin-colored to erythematous lesion May ulcarate	Basaloid cells with ductal differentiation Hyperkeratosis	Dilated vessels, structureless pink-whitish areas Ulceration Blue-gray nests	[13]
Amelanotic Melanoma	Asymmetrical macular lesions, with uniform pink or red coloration with peripheral brown pigment	Atypical melanocytes, absence of melanin pigment epithelioid cells	Irregular polymorphous vessels Blue-whitish structures Milky-red areas Scar-like depigmentation	[14,15]
Cutaneous Sarcoma: AFX	Rapidly enlarging, dome-shaped nodules Sun-exposed areas Eroded surface	Spindle cells arranged in a storiform pattern Infiltrating the subcutaneous tissues Poorly defined architecture	Red and white featureless regions Irregular linear vessels White areas	[16,17,18]
Cutaneous Sarcoma: CUPS	Subcutaneous nodule with a gradual enlargement Substantial size	Pleomorphic histiocyte-like and spindled cells Atypical mitotic activity	Red and white structureless areas Thick and irregular vessels	[16,17,18]
Aggressive Digital Papillary Adenocarcinoma	Painful nodular lesions on fingers prone to recurrence	Papillary structures Cuboidal or columnar cells Invasion into deep dermis tubuloalveolar and ductal structures Papillary projections extending into the cystic lumina	None or nonspecific findings, requires histopathology	[19]
Pseudoepitheliomatous Hyperplasia	Skin-colored or tan pink Well-demarcated plaque or nodule scaling and crusting	Hyperkeratosis, acanthosis pseudoepitheliomatous hyperplasia Exuberant growth of squamous epithelium with invagination into the dermis nodular masses, composed of sheets of cells and nests with large keratin pearls and keratin cysts no dysplasia	Scales, keratin, and white circles surrounding a dilated and plugged follicular infundibulum white structureless area Hairpin, linear, irregular, glomerular, or polymorphic vessels White homogenous area, along with keratotic follicular plugging surrounded by white circles	[20,21]
Lupus vulgaris	Soft, solitary plaque Brownish to reddish hue, advancing edge with scarring	Formation of tuberculoid granulomas With or without caseation surrounded by epithelioid Histiocytes and multinucleated giant cells	Linear focussed telangiectasias on a yellow-brown background white scales	[22,23]

AFX—Atypical Fibroxanthoma, CUPS—Cutaneous Undifferentiated Pleomorphic Sarcoma.

## Data Availability

No new data were created or analyzed in this study. Data sharing is not applicable to this article.
